# Dysfunctional Gut Microbiome Networks in Childhood IgE-Mediated Food Allergy

**DOI:** 10.3390/ijms22042079

**Published:** 2021-02-19

**Authors:** Khui Hung Lee, Jing Guo, Yong Song, Amir Ariff, Michael O’Sullivan, Belinda Hales, Benjamin J. Mullins, Guicheng Zhang

**Affiliations:** 1 School of Public Health, Curtin University of Technology, Bentley, WA 6102, Australia; khuihung.lee@postgrad.curtin.edu.au (K.H.L.); jing.guo2@postgrad.curtin.edu.au (J.G.); 2 The Menzies Institute for Medical Research, University of Tasmania, 17 Liverpool St, Hobart, TAS 7000, Australia; yong.song@utas.edu.au; 3 School of Women’s and Children’s Health, University of New South Wales, Sydney, NSW 2052, Australia; amir.ariff@unsw.edu.au; 4 Department of Immunology, Perth Children’s Hospital, Nedlands, WA 6009, Australia; 5 Telethon Kids Institute, University of Western Australia, West Perth, WA 6872, Australia; belinda.hales@telethonkids.org.au; 6 Curtin Health Innovation Research Institute, Curtin University, Kent St, Bentley, WA 6102, Australia; 7 Infection and Immunity, School of Biomedical Sciences, University of Western Australia, Crawley, WA 6000, Australia

**Keywords:** 16S rRNA gene sequencing, food allergy, microbiome, WGCNA, Ruminococcaceae

## Abstract

The development of food allergy has been reported to be related with the changes in the gut microbiome, however the specific microbe associated with the pathogenesis of food allergy remains elusive. This study aimed to comprehensively characterize the gut microbiome and identify individual or group gut microbes relating to food-allergy using 16S rRNA gene sequencing with network analysis. Faecal samples were collected from children with IgE-mediated food allergies (*n* = 33) and without food allergy (*n* = 27). Gut microbiome was profiled by 16S rRNA gene sequencing. OTUs obtained from 16S rRNA gene sequencing were then used to construct a co-abundance network using Weighted Gene Co-expression Network Analysis (WGCNA) and mapped onto Kyoto Encyclopedia of Genes and Genomes (KEGG) pathways. We identified a co-abundance network module to be positively correlated with IgE-mediated food allergy and this module was characterized by a hub taxon, namely *Ruminococcaceae UCG-002* (phylum Firmicutes). Functional pathway analysis of all the gut microbiome showed enrichment of methane metabolism and glycerolipid metabolism in the gut microbiome of food-allergic children and enrichment of ubiquinone and other terpenoid-quinone biosynthesis in the gut microbiome of non-food allergic children. We concluded that *Ruminococcaceae UCG-002* may play determinant roles in gut microbial community structure and function leading to the development of IgE-mediated food allergy.

## 1. Introduction

Emerging evidence has pointed towards the critical role of microbial communities in human health and disease, including regulation of the mucosal barrier function [1,2,3,4], metabolism [5,6,7] and host immune responses [3,4,8]. This is particularly evident in the gastrointestinal (GI) tract, where the diversity and richness of microorganisms are highest [9]. Changes in the gut microbiome commonly referred to as dysbiosis, may disrupt gut homeostasis and increase intestinal permeability, thereby causing immune system disorders such as autoimmune diseases and allergic disorders including food allergy [10,11,12].

Previous studies have started to unveil an association between the gut microbiome and the development of food allergy. A large observational cohort study in the United States showed that food-allergic children had a higher abundance of Bacteroidetes and a lower abundance of Firmicutes than children with resolved food allergy [13], while some studies showed the opposite results [14,15].

Considering the complexity of structure, function and compositional variability, the gut microbiome can be modelled and expressed as networks to infer the dynamic nature of the host–microbe interactions [16]. One approach to construct co-abundance network modules is to apply weighted gene co-expression network analysis (WGCNA) to quantify the co-abundance of operational taxonomic units (OTUs) across multiple samples. Developed by Horvath and colleagues, WGCNA was initially used to construct gene networks based on their similar biological functions and identify the hub gene that may associated with phenotypic traits [17]. We used WGCNA in this study to analyse the association between gut microbiome and disease phenotype by forming the complex microbial communities into different co-abundance network modules in order to identify hub taxa, the centralities of these co-abundance modules. Through this, we expect that WGCNA will identify potential target microbes, which may play a key role in regulating/influencing the microbe–microbe interactions, leading to the onset of food allergy.

## 2. Results

### 2.1. Gut Microbial Alpha Diversity

A total of 60 samples were included in our final analysis (33 food-allergic children and 27 non-food allergic children). Thirty-nine percent of the subjects were boys, with the median age for non-food allergic children and food-allergic children of 5.9 years and 5.0 years, respectively. The groups did not significantly differ from each other with regard to age (*p* = 0.200) and gender (*p* = 0.525). The food allergies noted in the food-allergic children included nuts (*n* = 23), egg (*n* = 4) and mixed allergies (*n* = 6).

To determine the average species diversity in a habitat or specific area, alpha diversity was evaluated using Chao1, Shannon index and observed OTUs matrices. Chao1 showed that non-food allergic children had lower species richness compared to food-allergic children, while Shannon index and observed OTUs showed that non-food allergic children and food-allergic children had similar gut microbial community richness and evenness (Table 1).

### 2.2. Gut Microbial Beta Diversity

To determine the degree of inter-group dissimilarity, beta diversity was evaluated using unweighted and weighted UniFrac distance matrices. Beta diversity did not show a significant difference between food-allergic children and non-food allergic children (Supplementary Figure S1).

### 2.3. Gut Microbial Composition

OTU dataset for food-allergic children and non-food allergic children consisted of 7 phyla, 14 classes, 16 orders, 28 families and 105 genera. At the phyla level, the gut microbiota was dominated by Firmicutes and Bacteroidetes, with lower abundance of Proteobacteria, Verrucomicrobia, Actinobacteria, Tenericutes and Cyanobacteria (Supplementary Figure S2) in children with and without food allergy. There was no significant difference in the phylum level between food-allergic children and non-food allergic children (Supplementary Table S1, Online Supplemental Notes).

One hundred and five genera were identified, and only 18 genera were accounted for more than 1% across all samples (Supplementary Table S2, Online Supplemental Notes). There was no significant difference in the genera level between food-allergic children and non-food allergic children.

### 2.4. Microbial Co-Abundance Network Modules

To better characterize gut microbial taxa in food-allergic children, we applied WGCNA to identify clusters of microbial taxa whose differential representation was correlated with food allergy. Each cluster was represented as a colour module.

Through WGCNA, we were able to identify 14 modules of co-abundant taxa and the number of taxa within modules ranged from 32 to 167 (Table 2). Among all the taxa, only 167 taxa (17%) were not included in any colour module, and these taxa were grouped into the grey module as per default.

### 2.5. Hub Taxa Associated with Food Allergy

The module eigengenes between children with and without food allergy were further compared with using module trait association analysis to identify the food allergy-associated modules.

**Figure 1:**
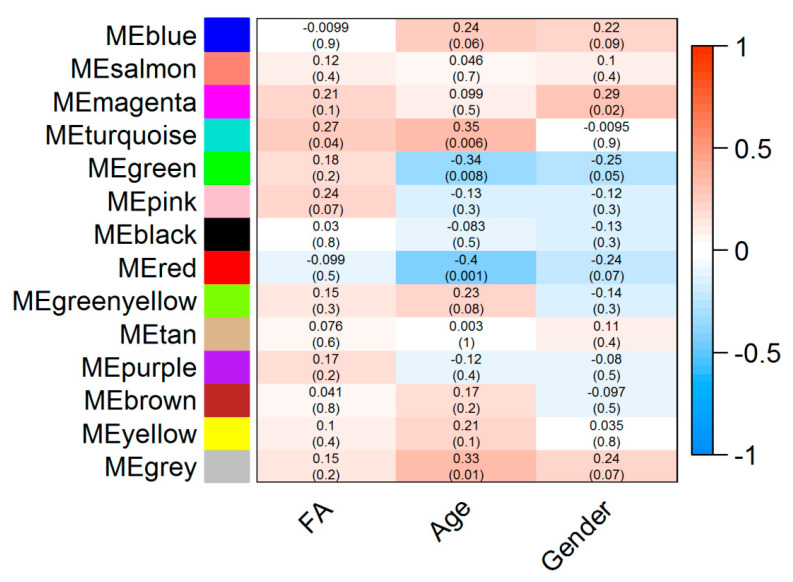
Module-trait associations. Each row corresponds to a module eigengene (ME) while each column corresponds to either phenotype (FA: Food allergy) or demographic traits such as age and gender. Each cell contains the corresponding correlation coefficient (display at the top of the cell) and corresponding p-values for each module (display at the bottom of the cells within parentheses). Blue and red colours of the spectrum on the right denote low and high correlation, respectively.

**Figure 2:**
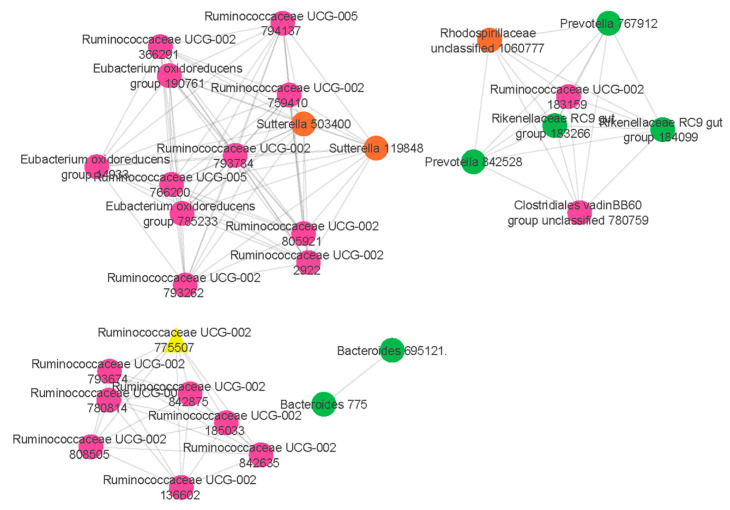
Network analysis identifies a distinct module of co-associated taxa. The highly correlated taxa in the comparisons of food allergic children and non-food allergic children are indicated and colour coded according to the phylum. Green colour represents Bacteroidetes phylum, pink colour represents Firmicutes phylum while orange colour represents Proteobacteria phylum. Hub taxon (yellow triangle) exhibits greatest intramodular connectivity, whereas connector taxa (circles) exhibit a higher frequency of intramodular connectivity.

Our results showed that a co-abundance network module (turquoise) was positively correlated with food allergy (*r* = 0.27 *p* = 0.04) (Figure 1). Particularly, *Ruminococcaceae UCG-002* was identified as the hub taxon (TaxaSignificance > 0.2 and Module Membership > 0.8) (Figure 2) for this module. In addition, 10 dominant taxa (>1% relative abundance across all samples) were also identified in the module. The majority of the dominant taxa came from Firmicutes phylum, including the genera of *Ruminococcaceae UCG-002*, *Eubacterium oxidoreducens group*, *Eubacterium coprostanoligenes group* and *Lachnospiraceae* (*NK4A136* and *UCG-008*). Other than this, the dominant taxa also included genera taxa from the phyla of Bacteroidetes (*Bacteroides*, *Alistipes*, *Parabacteroides* and *Prevotella 2*) as well as Proteobacteria (*Rhodospirillaceae*).

### 2.6. Predicted Functional Pathway of Gut Microbial Taxa Associated with Food Allergy

**Figure 3:**
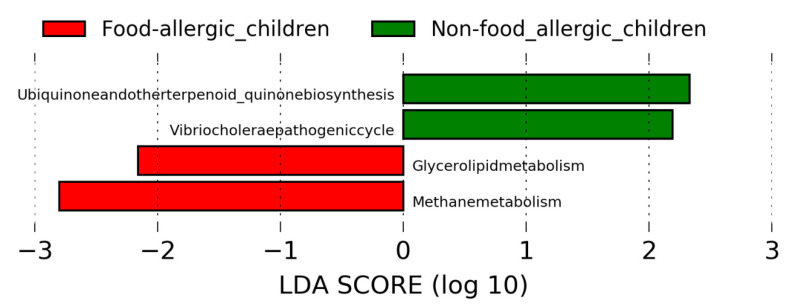
Linear discriminant analysis effect size (LEfSe) analysis revealed distinct KEGG pathway differences in gut microbiota between food-allergic children and non-food allergic children. KEGG pathway enriched in food-allergic children was indicated with red while the KEGG pathway enriched in non-food allergic children was indicated with green. Only the taxa that met a LDA significant threshold of >2 are displayed. LEfSe: Linear discriminant analysis effect size. LDA: Linear discriminant analysis. KEGG: Kyoto Encyclopedia of Genes and Genomes.

In order to have a better understanding of the functional pathway of gut microbial taxa that are associated with food allergy, linear discriminant analysis effect size (LEfSe) was performed by using the Tax4fun output. Using the threshold values (LDA > 2.0, *p* < 0.05), LEfSe revealed distinct KEGG pathway differences between gut microbiota of food-allergic children and non-food allergic children (Figure 3). Specifically, methane metabolism and glycerolipid metabolism were found to be enriched in food-allergic children. In contrast, ubiquinone and other terpenoid-quinone biosynthesis, as well as *Vibrio cholerae* pathogenic cycle were found to be enriched in non-food allergic children.

## 3. Discussion

There is increasing evidence that alterations in the gut microbiome are related to the development of food allergy [13,14,15,18], although the specific microbe associated with the pathogenesis of food allergy remains elusive. Our objective for this study was to perform 16S rRNA gene sequencing in integration with network analysis to characterize the gut microbiome and identify individual gut microbes or network modules of them that differ between food-allergic children and non-food allergic children. To our knowledge, this is the first study to characterize the gut microbiome of food-allergic children by applying network analysis.

Through network analysis, we identified a co-abundance network module (turquoise) to be positively correlated with food allergy and this module was characterized by a hub taxon, *Ruminococcaceae UCG-002* (Firmicutes phylum). It is suggested that a high relative abundance of *Ruminococcaceae* is associated with both food allergies [15], and a high fat diet in murine models [19,20,21], a factor which is known for its association with the development of food allergy. Taken together, these findings suggest that the high relative abundance of *Ruminococcaceae*, induced by a high fat diet, may produce acetic and propionic acid that possibly promote the synthesis of lipogenesis and cholesterol [22], which in turn dysregulated intestinal effector mast cell responses, as well as increased intestinal permeability and gut dysbiosis [23], leading to exacerbations of allergic responses.

We also identified a number of dominant taxa in this co-abundance network module that were highly related with food allergy, with the majority of them coming from phylum Firmicutes. Firmicutes has been suggested to play a role in modulating the immune system and subsequent development of allergic diseases [14,24]. A case-control study was conducted to investigate the association of gut microbiome and food allergy by comparing the gut microbiota composition between 34 infants with food allergy and 45 healthy controls [14]. The data revealed that the relative abundance of Firmicutes in food-allergic subjects was higher than that of the control subjects. Another study conducted by Chen et al. [24] also showed that Firmicutes was enriched in food-sensitized children.

The enrichment of pathways related to methane metabolism and glycerolipid metabolism (a subcategory of lipid metabolism) in the gut microbiome of food-allergic children was observed. However, KEGG pathways related to metabolism of cofactors and vitamins (ubiquinone and other terpenoid-quinone biosynthesis) was significantly enriched in the gut microbiome of non-food allergic children. Methane is the anaerobic fermentation product of endogenous and exogenous carbohydrates through intestinal microbiota [25]. The increase production of methane caused by high fat diet [26] may cause gastrointestinal disorders [25,27]. Our finding of enriched glycerolipid metabolism in food-allergic children was consistent with recognized roles of dietary lipid in regulating inflammation and food allergy [23,28]. A high-fat diet has been previously shown to change gut microbiota composition, leading to inflammation and food-allergic reactions. In contrast, the key role of ubiquinone in protecting against food allergy has been gaining attention lately. The deficiency of coenzyme Q10, which is a kind of ubiquinone, may develop and worsen the progress of food allergy in children [29].

Our finding of increased gut microbiota diversity in food-allergic children when compared with non-food allergic children appears contrary to several other food allergy studies, in which gut microbiota diversity was higher in healthy controls than food-allergic subjects. However, a study conducted by Fazlollahi et al. [15] has also shown that gut microbiota diversity could be higher in children with egg allergy compared to controls. Some other studies reported no association between gut microbiota diversity and food allergy [14,30]. This has indicated a subtle relationship between gut microbiota diversity and food allergy. Hence, the role of microbiome in food allergy was suggested to be considered along with the interplay between different taxa and their metabolic effects rather than only examining a single dimension, bacterial diversity.

Taken together, we speculate that increased abundance of *Ruminococcaceae* along with other dominant microbial taxa, may remodel the normal gut microbial ecosystem into a state of dysbiosis through the pathways of methane metabolism and glycerolipid metabolism, which in turn elicit a host IgE-mediated allergic response. Our findings highlight the usefulness of network analysis in disentangling the hub taxon, *Ruminococcaceae* that play determinant roles in gut microbial community structure and functions leading to IgE-mediated food allergy. The differences in the co-abundance patterns of gut microbiome between children with and without food allergy can help us understand the complex interrelationships between gut microbiome and pathogenesis of food allergies. This information potentially aids targeted dietary or probiotic strategies for clinical practice to improve food allergy outcomes. Although our study revealed there was an association between gut microbiome network and development of food allergy, there were several limitations in the study. Firstly, the sample size was small. However, the application of network analysis in our study has deciphered key microbial populations that may be associated with food allergy, including those with low relative abundance but highly relevant to the onset of food allergy through characterizing the interactions of microbes at the community scale. Secondly, 16S rRNA gene sequencing is only sensitive to the genus level, but not species and strains. Thirdly, as this was a cross-sectional study, our results could not indicate a causal relationship between the gut microbiome and food allergy. Finally, as our study aimed to construct a microbial network through 16S rRNA gene sequencing and weighted correlation network analysis, the actual roles of these taxa predicted to be related to food allergy have not yet been evaluated. Therefore, further studies utilizing metagenomic analysis or real-time PCR in larger cohorts are required to confirm our results.

## 4. Materials and Methods

### 4.1. Study Subjects

From January 2018 to March 2019, children with immunologist-diagnosed food allergy, were recruited from Immunology Outpatient Clinic, Perth Children’s Hospital. Children from 1 year old to 7 years of age with immunologist-diagnosed food allergy were eligible for participation. Non-food allergic children, with age and gender matched were recruited from the local community.

All parents of the subjects gave their informed consent for inclusion before they participated in the study. The study was conducted in accordance with the National Health and Medical Research Council National Statement on Ethical Conduct in Human Research, and the protocol was approved by the Human Research Ethics Committee (HREC), Perth Children’s Hospital (RGS151/HREC 2017060EP) and Curtin University (HRE2017-0712).

### 4.2. Faecal Sample Collection and Processing

Parents/guardians of the participants were provided a faecal collection kit, which included a protocol of faecal collection, a screw cap faecal container (Sarstedt, Germany), an underpad sheet, a pair of disposable gloves, a white paper bag and a sealed plastic bag with labels. Once collected, the faecal sample would then be transported on ice by a researcher within 2 hours of collection to the laboratory −80 °C freezers for storage.

DNA was then extracted using the QIAamp DNA Stool Mini Kit (Qiagen, Germany) in accordance with the manufacturer’s instructions. The PCR amplication and sequencing of sixty stool samples were performed by Novogene Bioinformatics Technology Co., Ltd. (Beijing, China). Briefly, PCR was carried out using Phusion^®^ High-Fidelity PCR Master Mix and GC Buffer (New England Biolabs, Beijing, China) in accordance with the manufacturer’s instruction. PCR thermal cycling was set as follows: initial denaturation at 98 °C for 1min, followed by 35 cycles at 98 °C for 10 s, 50 °C for 30 s and 72 °C for 90 s, and a final extension at 72 °C for 5 min. The samples were then subjected to electrophoresis on a 2% agarose gel for detection. Samples with a bright main strip between 400 and 450 bp were chosen for further analysis. The PCR products were purified using the Gene JET Gel Extraction kit (Thermo Scientific), and the sequencing libraries were constructed using Ion Plus Fragment Library Kit (Thermo Fisher Scientific, USA) in accordance with the manufacturer’s instruction. The library quality was monitored using a Qubit 2.0 Fluorometer (Thermo Fisher Scientific, St. Louis, MO, USA) and a Bioanalyzer 2100 system (Agilent Technologies, Santa Clara, CA, USA). Lastly, the library, which targeted the V3–V4 region of the 16S rRNA gene was sequenced on the Ion S5 XL platform (Thermo Fisher). A total of 4,858,507 sequences reads that passed the quality check (>Q20, error rate < 1%) were generated.

### 4.3. Quantitative Insights into Microbial Ecology (QIIME)

The raw sequences were then demultiplexed and quality filtered using QIIME [31] by removing those raw sequences with read-quality score less than 19, setting length fall below 3bp and consecutive quality base below 75%. The filtered sequences were then screened for chimeras using the usearch61 algorithm [32] and putative chimeric sequences were removed from the dataset. Sequences were clustered into operational taxonomic units (OTUs) at a 97% similarity level against the SILVA reference database (release 128) [33]. The OTUs with low relative abundance (less than 0.005%) were removed. All further analyses were performed at a rarefied depth of 22,178 sequences per sample to correct for differences in the read depth across samples. Alpha diversity and beta diversity of microbial communities were analysed using QIIME. Alpha diversity was estimated using two different indices: (1) Chao1, which takes into accounts only the abundance; (2) observed OTUs, which takes into accounts only the observed species; (3) the Shannon index, which takes into accounts the abundance and evenness of OTUs. Beta diversity was measured using the weighted and unweighted UniFrac distance matrices. Principal Coordinate Analysis (PCoA) was obtained to visualise unweighted and weighted Unifrac distances in a two-dimensional structure. The Adonis permutational multivariate analysis (Adonis/PERMANOVA) was performed to compare beta diversity dissimilarity matrices. A comparison of the relative abundance of OTUs between groups was computed using the Mann–Whitney test. A probability value of *p* < 0.05 was considered statistically significant.

### 4.4. Construction of Microbial Co-Abundance Network

In order to have a better understanding of the co-abundance network of the microbial taxa, Weighted Gene Correlation Network Analysis (WGCNA) package of R [17] was then performed to conduct network analysis by using OTU count data (with 97% identity threshold), which has undergone Hellinger transformation, by transforming OTU count data from absolute to relative abundance that gives low weights to variables with low counts and many zeros [34].

Taking into account that the use of correlation analysis in analysing the microbiome data can lead to a spurious association, WGCNA applied few steps to reduce the number of false positive connections introduced by spurious associations [17]. A soft thresholding power β was determined based on scale-free topology index (R2) of 0.85. The most appropriate soft thresholding power was then used to construct a weighted adjacency matrix to which the co-abundance similarity has been raised. By raising the absolute value of the correlation to a soft thresholding power (β ≥ 1), this step emphasized a strong correlation coefficient. Then, to further minimize the effects of noise and spurious associations, the adjacency matrix was transformed into a topological overlap matrix and the corresponding dissimilarity was calculated. This topological overlap matrix was particularly useful when the original adjacency matrix was sparse or susceptible to noise by replacing the isolated connections with weighted neighbourhood overlaps, thus, reducing the effects of spurious associations leading to a more robust network. The modules were subsequently identified using a dynamic tree cut algorithm with a minimum cluster size of 30 and merge cut height of 0.25 and later assigned the clusters of highly co-occurred taxa to different colours for visualization.

After that, module trait association analysis was used to calculate the correlation coefficient between modules and food allergy as well as demographics traits such as age and gender. Modules with *p* values < 0.05 were regarded significant food allergy-related modules.

### 4.5. Hub Taxa Selection and Visualization

Next, an intramodular analysis was performed to determine the hub taxa by summing the connection strengths with other module taxa. Moreover, the hub taxa have to meet the absolute value of the TaxaSignificance > 0.2 and Module Membership > 0.8. Taxa of the significant modules were then visualized using Cytoscape v3.8.0 [35].

### 4.6. Kyoto Encyclopedia of Genes and Genomes (KEGG) Pathway Analysis

All OTUs table and OTUs taxonomy were mapped onto Kyoto Encyclopedia of Genes and Genomes (KEGG) pathways using R package, Tax4Fun. Linear discriminant analysis (LDA) effect size (LEfSe) analysis (http://huttenhower.sph.harvard.edu/lefse/ (accessed on 18 February 2021)) was performed to detect biomarkers of the Kyoto Encyclopedia of Genes and Genomes (KEGG) pathways that differed significantly between non-food allergic children and food-allergic children. Default settings (alpha = 0.05, effect-size threshold of 2) were applied.

## 5. Conclusions

Our study provides a better understanding of the gut microbiome with respect to the presence of *Ruminococcaceae UCG-002* interacting with other dominant taxa including *Eubacterium oxidoreducens group*, *Eubacterium coprostanoligenes group*, *Lachnospiraceae* (*NK4A136* and *UCG-008*), *Bacteroides*, *Alistipes*, *Parabacteroides*, *Prevotella 2* as well as *Rhodospirillaceae* in the pathogenesis of IgE-mediated food allergy and these microbial taxa were mainly involved in methane metabolism and glycerolipid metabolism. Integrative view of gut microbial ecology based on the microbial module in our study may help to understand the microbial interactions associated with IgE-mediated food allergy.

## Figures and Tables

**Table 1:** Comparison of gut microbial alpha diversity between food-allergic children and non-food allergic children. Values represent mean ± SD.

Alpha Diversity	Non-Food Allergic Children	Food-Allergic Children	*p*
Chao1	565.7 ± 91.7	622.3 ± 87.4	**0.02**
Observed OTUs	458.9 ± 86.0	502.8 ± 83.9	0.058
Shannon diversity index	5.3 ± 0.7	5.5 ± 0.7	0.395

Bold value indicates a statistically significant difference with a *p*-value less than 0.05.

**Table 2:** The number of taxa in the 14 modules.

Module Colours	Frequency
Black	54
Blue	88
Brown	88
Green	67
green-yellow	34
Grey	167
Magenta	48
Pink	51
Purple	47
Red	66
Salmon	32
Tan	33
Turquoise	114
Yellow	82

## Data Availability

All raw sequencing reads are available at NCBI BioProject database (PRJNA699997) (https://www.ncbi.nlm.nih.gov/bioproject/, accessed on 18 February 2021).

## Supplementary Materials

The following are available online at https://www.mdpi.com/1422-0067/22/4/2079/s1, Supplementary Figure S1: PCoA plots of individual gut microbiota in food-allergic children (red) and non-food allergic children (blue) derived from (a) unweighted and (b) weighted UniFrac distances. Each symbol represents a sample. PCoA: Principal Coordinate Analysis, Supplementary Figure S2: Relative abundance of gut microbial phyla, Supplementary Table S1: The comparison of gut microbiota at the phyla level between food-allergic children and non-food allergic children, Supplementary Table S2: Relative abundance of predominant genera in gut microbiota between food-allergic children and non-food allergic children (≥1% across all samples), Online Supplemental Notes.

## Abbreviations

LDA LEfSe OTUs	Linear discriminant analysis Linear discriminant analysis effect size Operational Taxonomic Units
ME	Module Eigengene
PCoA QIIME	Principal Coordinate Analysis Quantitative Insights Into Microbial Ecology
WGCNA	Weighted Gene Correlation Network Analysis

## References

[1] Martens E.C., Neumann M., Desai M.S. (2018). Interactions of commensal and pathogenic microorganisms with the intestinal mucosal barrier. Nat. Rev. Genet..

[2] Kuhn K.A., Pedraza I., Demoruelle M.K. (2014). Mucosal Immune Responses to Microbiota in the Development of Autoimmune Disease. Rheum. Dis. Clin. N. Am..

[3] Kamada N., Chen G.Y., Inohara N., Núñez G. (2013). Control of pathogens and pathobionts by the gut microbiota. Nat. Immunol..

[4] Hooper L.V., Macpherson A.J. (2010). Immune adaptations that maintain homeostasis with the intestinal microbiota. Nat. Rev. Immunol..

[5] Wahlström A., Sayin S.I., Marschall H.-U., Bäckhed F. (2016). Intestinal Crosstalk between Bile Acids and Microbiota and Its Impact on Host Metabolism. Cell Metab..

[6] Nieuwdorp M., Gilijamse P.W., Pai N., Kaplan L.M. (2014). Role of the Microbiome in Energy Regulation and Metabolism. Gastroenterology.

[7] Joyce S.A., Gahan C.G. (2014). The gut microbiota and the metabolic health of the host. Curr. Opin. Gastroenterol..

[8] Honda K., Littman D.R. (2016). The microbiota in adaptive immune homeostasis and disease. Nat. Cell Biol..

[9] Lozupone C.A., Stombaugh J.I., Gordon J.I., Jansson J.K., Knight R. (2012). Diversity, stability and resilience of the human gut microbiota. Nat. Cell Biol..

[10] Levy M., Kolodziejczyk A.A., Thaiss C.A., Elinav E. (2017). Dysbiosis and the immune system. Nat. Rev. Immunol..

[11] Ihekweazu F.D., Versalovic J. (2018). Development of the Pediatric Gut Microbiome: Impact on Health and Disease. Am. J. Med. Sci..

[12] Das B., Nair G.B. (2019). Homeostasis and dysbiosis of the gut microbiome in health and disease. J. Biosci..

[13] Bunyavanich S., Shen N., Grishin A., Wood R., Burks W., Dawson P., Jones S.M., Leung D.Y., Sampson H., Sicherer S. (2016). Early-life gut microbiome composition and milk allergy resolution. J. Allergy Clin. Immunol..

[14] Ling Z., Li Z., Liu X., Cheng Y., Luo Y., Tong X., Yuan L., Wang Y., Sun J., Li L. (2014). Altered fecal microbiota composi-tion associated with food allergy in infants. Appl. Environ. Microbiol..

[15] Fazlollahi M., Chun Y., Grishin A., Wood R.A., Burks A.W., Dawson P., Jones S.M., Leung D.Y., Sampson H.A., Sicherer S.H. (2018). Early-life gut microbiome and egg allergy. Allergy.

[16] Layeghifard M., Hwang D.M., Guttman D.S. (2017). Disentangling Interactions in the Microbiome: A Network Perspective. Trends Microbiol..

[17] Langfelder P., Horvath S. (2008). WGCNA: An R package for weighted correlation network analysis. BMC Bioinform..

[18] Goldberg M., Gershon H., Appel M., Nachshon L., Levy M.B., Youngster I., Elizur A., Koren O. (2019). Distinctive Gut Microbiota Signature in Persistent IgE-mediated Food Allergy. J. Allergy Clin. Immun..

[19] Wang Z., Lam K., Hu J., Ge S., Zhou A., Zheng B., Zeng S., Lin S. (2019). Chlorogenic acid alleviates obesity and modulates gut microbiota in high-fat-fed mice. Food Sci. Nutr..

[20] Liu Z., Wang N., Ma Y., Wen D. (2019). Hydroxytyrosol Improves Obesity and Insulin Resistance by Modulating Gut Microbiota in High-Fat Diet-Induced Obese Mice. Front. Microbiol..

[21] Kim K.-A., Gu W., Lee I.-A., Joh E.-H., Kim D.-H. (2012). High Fat Diet-Induced Gut Microbiota Exacerbates Inflammation and Obesity in Mice via the TLR4 Signaling Pathway. PLoS ONE.

[22] Kieler I.N., Kamal S.S., Vitger A.D., Nielsen D.S., Lauridsen C., Bjornvad C.R. (2017). Gut microbiota composition may relate to weight loss rate in obese pet dogs. Vet. Med. Sci..

[23] Hussain M., Bonilla-Rosso G., Chung C.K.K., Bäriswyl L., Rodriguez M.P., Kim B.S., Engel P., Noti M. (2019). High dietary fat intake induces a microbiota signature that promotes food allergy. J. Allergy Clin. Immunol..

[24] Chen C.-C., Chen K.-J., Kong M.-S., Chang H.-J., Huang J.-L. (2016). Alterations in the gut microbiotas of children with food sensi-tization in early life. Pediatr. Allergy Immunol..

[25] Monasta L., Pierobon C., Princivalle A., Martelossi S., Marcuzzi A., Pasini F., Perbellini L. (2017). Inflammatory bowel disease and patterns of volatile organic compounds in the exhaled breath of children: A case-control study using Ion Molecule Reaction-Mass Spectrometry. PLoS ONE.

[26] Mathur R., Kim G., Morales W., Sung J., Rooks E., Pokkunuri V., Weitsman S., Barlow G.M., Chang C., Pimentel M. (2013). Intestinal Methanobrevibacter smithii but not total bacteria is related to diet-induced weight gain in rats. Obesity.

[27] Blais Lecours P., Marsolais D., Cormier Y., Berberi M., Hache C., Bourdages R., Duchaine C. (2014). Increased prevalence of Meth-anosphaera stadtmanae in inflammatory bowel diseases. PLoS ONE.

[28] López-Fandiño R. (2019). Role of dietary lipids in food allergy. Crit. Rev. Food Sci. Nutr..

[29] Miles M.V., Putnam P.E., Miles L., Tang P.H., DeGrauw A.J., Wong B.L., Horn P.S., Foote H.L., Rothenberg M.E. (2011). Ac-quired coenzyme Q10 deficiency in children with recurrent food intolerance and allergies. Mitochondrion.

[30] Savage J.H., Lee-Sarwar K.A., Sordillo J., Bunyavanich S., Zhou Y., O’Connor G., Sandel M., Bacharier L.B., Zeiger R., Sodergren E. (2018). A prospective microbiome-wide association study of food sensitization and food allergy in early childhood. Allergy.

[31] Caporaso J.G., Kuczynski J., Stombaugh J., Bittinger K., Bushman F.D., Costello E.K., Fierer N., Peña A.G., Goodrich J.K., I Gordon J. (2010). QIIME allows analysis of high-throughput community sequencing data. Nat. Methods.

[32] Edgar R.C. (2010). Search and clustering orders of magnitude faster than BLAST. Bioinformatics.

[33] Quast C., Pruesse E., Yilmaz P., Gerken J., Schweer T., Yarza P., Peplies J., Glockner F.O. (2013). The SILVA ribosomal RNA gene database project: Improved data processing and web-based tools. Nucleic Acids Res..

[34] Wilson J.M., Litvin S.Y., Beman J.M. (2018). Microbial community networks associated with variations in community respiration rates during upwelling in nearshore Monterey Bay, California. Environ. Microbiol. Rep..

[35] Cline M.S., Smoot M., Cerami E., Kuchinsky A., Landys N., Workman C., Christmas R., Avila-Campilo I., Creech M., Gross B. (2007). Integration of biological networks and gene expression data using Cytoscape. Nat. Protoc..

