# Supplementary figures and images for: Dysfunctional Gut Microbiome Networks in Childhood IgE-Mediated Food Allergy
Source: Int J Mol Sci. 2021 Feb 19;22(4):2079. doi: 10.3390/ijms22042079 (PMC7923212; doi:10.3390/ijms22042079)

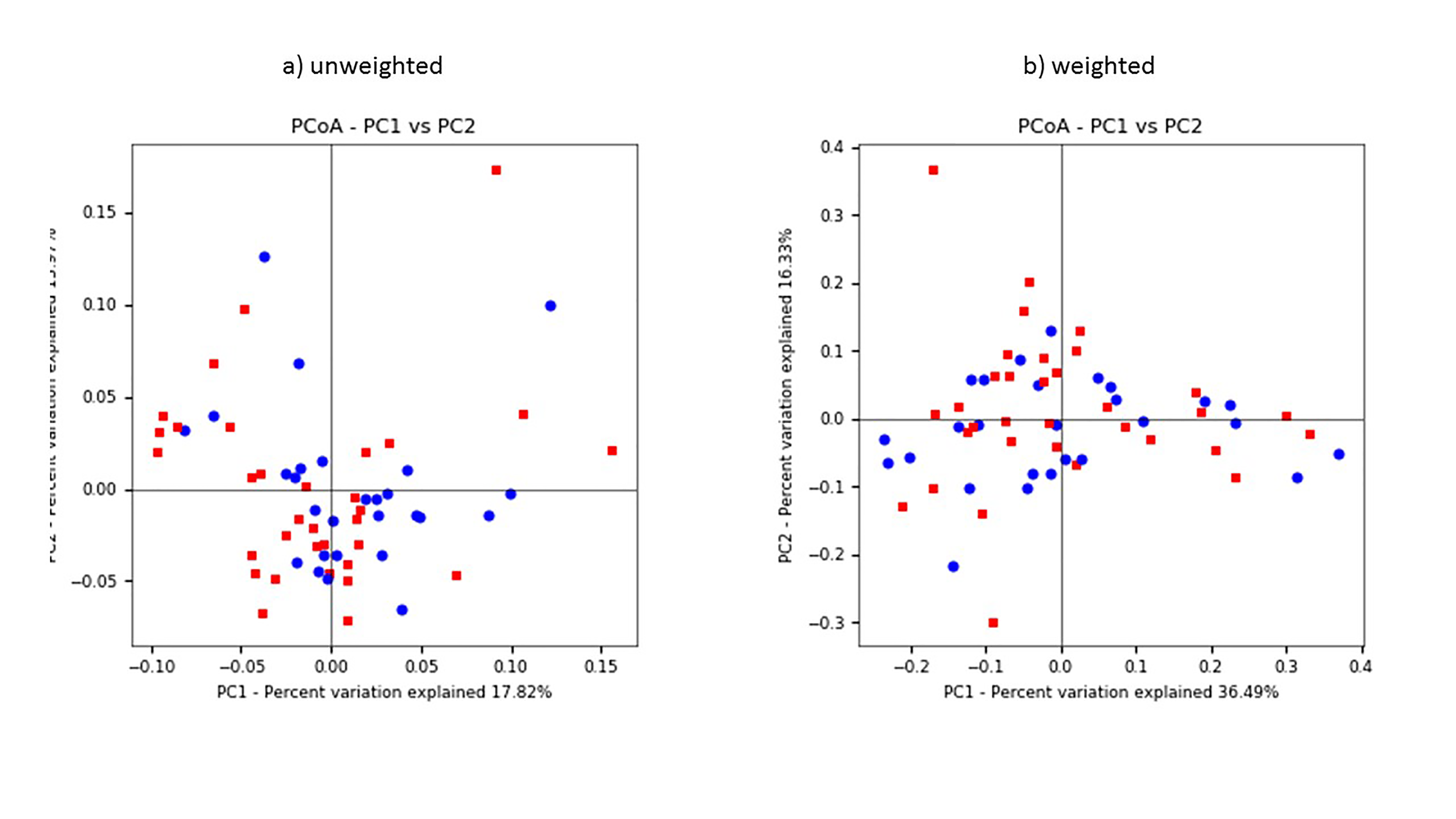

Supplement: Supplementary file 1 [file ijms-22-02079-s001.zip › Supplementary Figure S1.tif]

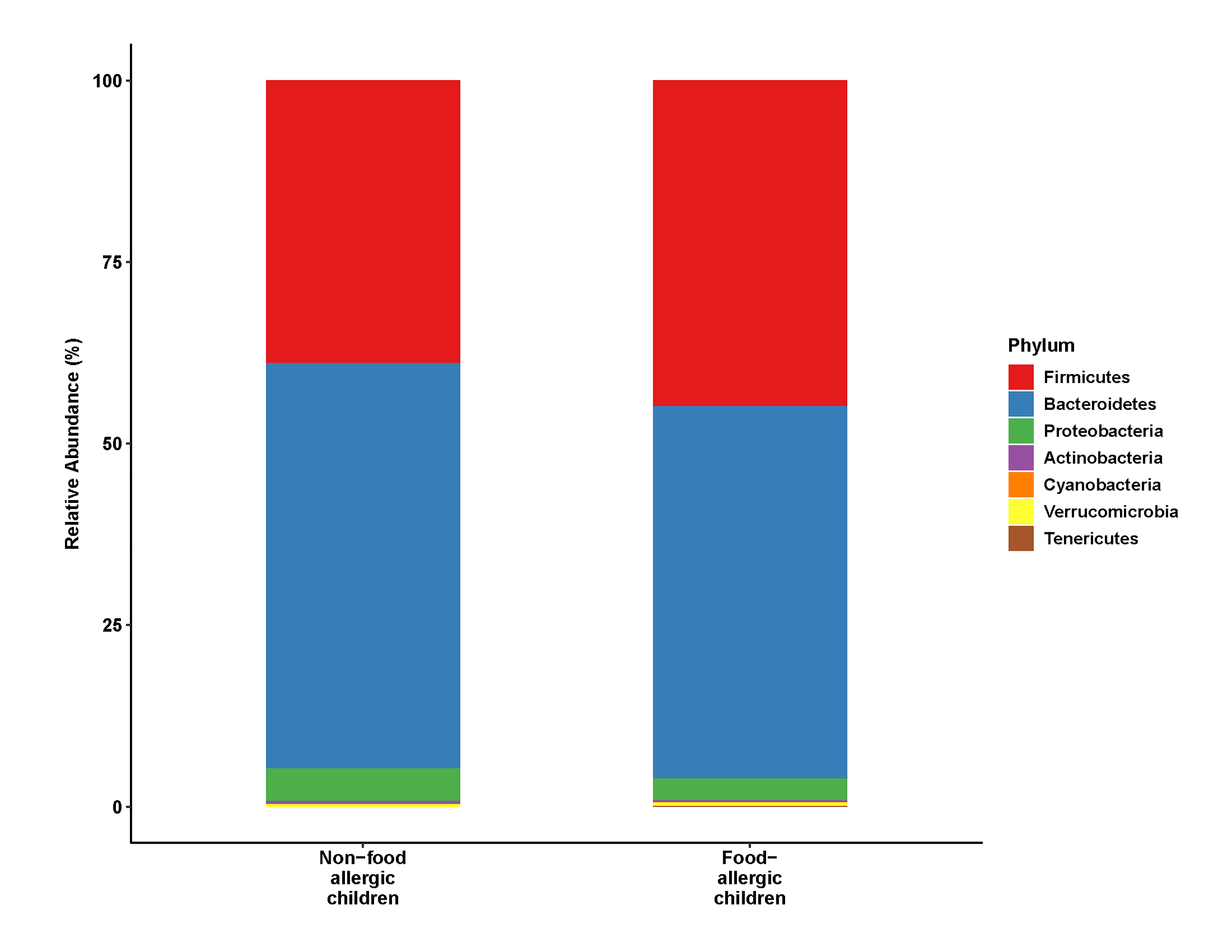

Supplement: Supplementary file 1 [file ijms-22-02079-s001.zip › Supplementary Figure S2.tiff]
