# Supplementary material for: Dysfunctional Gut Microbiome Networks in Childhood IgE-Mediated Food Allergy
Source: Int J Mol Sci. 2021 Feb 19;22(4):2079. doi: 10.3390/ijms22042079 (PMC7923212; doi:10.3390/ijms22042079)
Supplement: Supplementary file 1 [file ijms-22-02079-s001.zip › Online Supplemental Notes.doc]

**Online Supplemental Notes**

**Results:**

**Gut microbial composition**

Food-allergic children had a higher relative abundance of Firmicutes, Tenericutes, Verrucomicrobia and Cyanobacteria and a lower relative abundance of Bacteroidetes Proteobacteria, and Actinobacteria than non-food allergic children (Supplementary Table S1).

Although there were no statistically significant differences between the two groups; there was a trend toward lower read counts for *Bacteroides**, Prevotella 9*, *Allistipes*, *Parabacteroides*, *Faecalibacterium,* *Lachnospiraceae UCG-008, Ruminococcus 1,* and *Parasutterella* in those with food allergy compared to those without food allergy. In contrast, there was a trend toward higher read counts for *Ruminococcaceae UCG-002*, *Eubacterium eligens group*, *Subdoligranulum*, *Barnesiella*, *Roseburia* and *Eubacterium coprostanoligenes group* in food-allergic children as compared to non-food allergic children (Supplementary Table S2).
