# Supplementary material for: Dysfunctional Gut Microbiome Networks in Childhood IgE-Mediated Food Allergy
Source: Int J Mol Sci. 2021 Feb 19;22(4):2079. doi: 10.3390/ijms22042079 (PMC7923212; doi:10.3390/ijms22042079)
Supplement: Supplementary file 1 [file ijms-22-02079-s001.zip › Supplementary Figures.doc]

Supplementary Figure S1. PCoA plots of individual gut microbiota in food-allergic children (red) and non-food allergic children (blue) derived from (a) unweighted and (b) weighted UniFrac distances. Each symbol represents a sample. PCoA: Principal Coordinate Analysis.

Supplementary Figure S2. Relative abundance of gut microbial phyla.
