# Supplementary material for: Dysfunctional Gut Microbiome Networks in Childhood IgE-Mediated Food Allergy
Source: Int J Mol Sci. 2021 Feb 19;22(4):2079. doi: 10.3390/ijms22042079 (PMC7923212; doi:10.3390/ijms22042079)
Supplement: Supplementary file 1 [file ijms-22-02079-s001.zip › Supplementary Tables.doc]

| **Supplementary Table S1. The comparison of gut microbiota at the phyla level between food-allergic children and non-food allergic children.** | | | | |
| --- | --- | --- | --- | --- |
| **Phylum** | **Non-food allergic children** | **Food-allergic children** | ***p*** |  |
| Proteobacteria | 4.55 | 3.08 | 0.071 |  |
| Tenericutes | 0.01 | 0.17 | 0.127 |  |
| Firmicutes | 38.9 | 44.84 | 0.169 |  |
| Actinobacteria | 0.35 | 0.26 | 0.231 |  |
| Bacteroidetes | 55.77 | 51.19 | 0.274 |  |
| Verrucomicrobia | 0.38 | 0.41 | 0.312 |  |
| Cyanobacteria | 0.01 | 0.02 | 0.510 |  |

Values expressed as relative abundance (%).

*p*: p value.

| **Supplementary Table S2. Relative abundance of predominant genera in gut microbiota between food-allergic children and non-food allergic children (≥ 1% across all samples)** | | | | | | | | |
| --- | --- | --- | --- | --- | --- | --- | --- | --- |
| **Genus** | **Non-food allergic children** | | **Food-allergic children** | | ***p*** | |  | |
| Ruminococcaceae UCG-002 | | 1.33 | | 3.41 | | 0.071 | |  |
| Eubacterium oxidoreducens group | | 0.74 | | 1.15 | | 0.073 | |  |
| Prevotella 9 | | 7.74 | | 5.84 | | 0.155 | |  |
| Eubacterium eligens group | | 1.2 | | 1.63 | | 0.193 | |  |
| Alistipes | | 6.45 | | 5.58 | | 0.209 | |  |
| Subdoligranulum | | 1.7 | | 2.69 | | 0.243 | |  |
| Faecalibacterium | | 11.56 | | 10.26 | | 0.268 | |  |
| Barnesiella | | 1.19 | | 1.41 | | 0.323 | |  |
| *Lachnospiraceae NK4A136 group* | | 0.83 | | 1.47 | | 0.353 | |  |
| Ruminococcus 1 | | 1.39 | | 1.11 | | 0.368 | |  |
| Lachnospiraceae UCG-008 | | 5.91 | | 5.28 | | 0.409 | |  |
| Roseburia | | 2.11 | | 3.34 | | 0.444 | |  |
| Lachnospira | | 0.65 | | 1.32 | | 0.518 | |  |
| Eubacterium coprostanoligenes group | | 1.4 | | 1.46 | | 0.682 | |  |
| Sutterella | | 1.26 | | 0.86 | | 0.783 | |  |
| Bacteroides | | 35.13 | | 34.05 | | 0.899 | |  |
| Parasutterella | | 1.87 | | 1.35 | | 0.958 | |  |
| Parabacteroides | | 3.8 | | 2.88 | | 0.994 | |  |

Values expressed as relative abundance (%).

*p*: p value.
